# Case report: Systemic tuberculosis with prostate involvement mimicking prostate cancer with multiple metastases on ^18^F-FDG and ^18^F-PSMA PET/CT

**DOI:** 10.3389/fmed.2024.1430300

**Published:** 2024-08-14

**Authors:** Xinyao Sun, Yongkang Qiu, Lele Song, Lei Kang

**Affiliations:** Department of Nuclear Medicine, Peking University First Hospital, Beijing, China

**Keywords:** prostate, tuberculosis, ^18^F-FDG, ^18^F-PSMA, PET/CT

## Abstract

**Background:**

Prostate tuberculosis is a common form of urogenital tuberculosis that occurs in men. Clinical and imaging manifestations of prostate tuberculosis are atypical, which often need to be differentiated from benign prostatic hyperplasia, a prostate malignant tumor, and a urinary tract infection. Although prostate-specific membrane antigen (PSMA) is considered a specific biomarker for prostate cancer, it is also found within tuberculosis tissues that may be stimulated by angiogenic factors. An abnormal PSMA uptake on positron emission tomography combined with computed tomography (PET/CT) should eliminate the possibility of tuberculosis.

**Case report:**

In this study, we reported a case of a 51-year-old man with an elevated erythrocyte sedimentation rate (ESR) but a normal prostate-specific antigen (PSA) value. 2-Deoxy-2-[fluorine-18]-fluoro-D-glucose (^18^F-FDG) and [fluorine-18]-prostate-specific membrane antigen (^18^F-PSMA) PET/CT scans were performed for further evaluation. The prostate showed a high fluoro-D-glucose (FDG) uptake but a slight PSMA uptake. Multiple osteolytic bone destruction and lymph nodes with an increased FDG uptake but a mild PSMA uptake were observed throughout the body. Systemic tuberculosis was diagnosed based on the prostate biopsy and the positive result of the T-cell spot test regarding tuberculosis infection. After 6 months of standard anti-tuberculosis treatment, the patient experienced symptom relief.

**Conclusion:**

In the case of a urinary tract infection, where the prostate shows high FDG uptake lesions with perilesional abscess, a mildly increased PSMA uptake, a low PSA value, a high ESR, and relevant clinical symptoms, tuberculosis should be considered and laboratory tests are required, especially when symptoms are relieved after successful anti-tuberculosis therapy. The final confirmation of the diagnosis still relies on pathological examination.

## Introduction

Among extrapulmonary tuberculosis (EPTB) forms, urogenital tuberculosis ranks third after lymph node involvement and tuberculosis pleural effusion (1). The incidence of prostate tuberculosis is 6.6% of urogenital tuberculosis (2). It is primarily caused by distant lesions that spread to the prostate via the bloodstream. Meanwhile, it may also spread from renal tuberculosis. Symptoms and imaging examinations of prostate tuberculosis are atypical, which often need to be differentiated from a prostate malignant tumor. Prostate-specific membrane antigen (PSMA) is an ideal diagnostic and therapeutic target for prostate cancer since it has low levels of expression in normal prostate tissues and non-prostate cancer tissues. In this study, we reported a case of a 51-year-old man with systemic tuberculosis involving the prostate, who had a high erythrocyte sedimentation rate (ESR) but a normal Prostate-specific antigen (PSA) value. His prostate showed a high fluoro-D-glucose (FDG) uptake but a slight PSMA uptake during positron emission tomography combined with computed tomography (PET/CT).

### Case report

**Figure 1:**
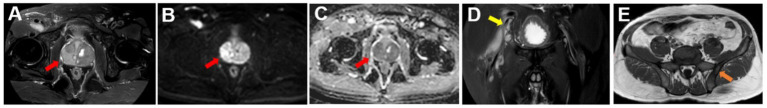
MRI imaging. The prostate lesions showed heterogeneous intensity on T2WI **(A)**, heterogeneous hyperintensity on DWI **(B)**, and an apparent diffusion coefficient (ADC) with hypointensity **(C)**. A lesion with hyperintensity on T2WI around the right iliopsoas muscle **(D)**. Bilateral iliac bone destruction on T1WI **(E)**.

A 51-year-old male presented with night sweats, loss of appetite, and 10 kg weight loss over the past 6 months. He had difficulty urinating for more than 3 months. Therefore, he came to the hospital for laboratory tests and imaging investigations of the urinary system. Some important dates and times, as well as the magnetic resonance imaging (MRI), 2-Deoxy-2-[fluorine-18]-fluoro-D-glucose (^18^F-FDG), and [fluorine-18]-prostate-specific membrane antigen (^18^F-PSMA) of the patient, are shown in Supplementary Figure S1. The laboratory tests revealed a high ESR of 77 mm/h (normal range, 0-15 mm/h), normal free prostate-specific antigen (f-PSA) of 0.151 ng/mL and total prostate-specific antigen (t-PSA) of 1.900 ng/mL, and slightly high neuron-specific enolase (NSE) of 20.19 ng/mL (normal range, <16.3 ng/mL). The MRI showed prostate lesions with isointensity on T1-weighted imaging (T1WI), heterogeneous intensity on T2-weighted imaging (T2WI) (Figure 1A), heterogeneous hyperintensity on diffusion-weighted imaging (DWI) (Figure 1B), and an apparent diffusion coefficient (ADC) with hypointensity (Figure 1C). A lesion with hyperintensity on T2WI was observed around the right iliopsoas muscle (Figure 1D). Bilateral iliac bone destruction and multiple swollen lymph nodes adjacent to the right iliac vessels were also found (Figure 1E). The possibility of prostate cancer with bone metastasis was considered.

**Figure 2:**
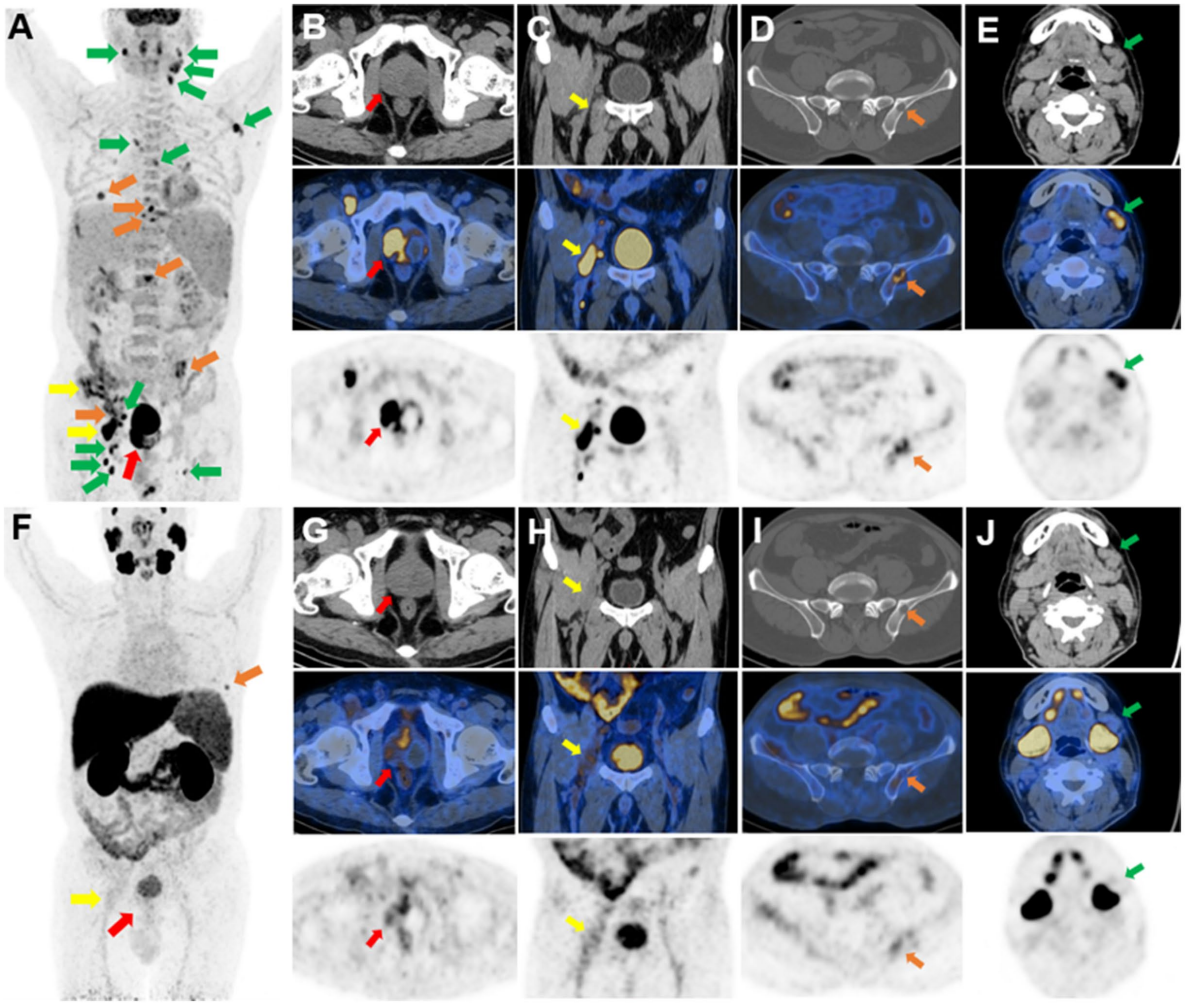
^18^F-FDG and ^18^F-PSMA PET/CT imaging. Multiple FDG-avid lesions **(A)** on PET MIP images. An unevenly increased FDG uptake on the right side of the prostate, with an SUV_max_ of 17.6 **(B)**. A high FDG uptake in a low-density lesion on the right iliopsoas muscle, with an SUV_max_ of 18.6 **(C)**. Destruction of the left iliac bone with an increased FDG uptake **(D)**. Multiple lymph nodes of the left neck with a high FDG uptake **(E)**. Multiple PSMA-avid lesions **(F)** on PET MIP images. A slightly unevenly increased PSMA uptake in the prostate **(G)**, the low-density lesion of the right iliopsoas muscle **(H)**, bones **(I)**, and lymph nodes **(J)**.

^18^F-FDG and ^18^F-PSMA PET/CT were performed to further evaluate the overall condition. ^18^F-PSMA was developed through the method of ^18^F-aluminum fluoride (Al^18^F) labeling in our hospital (3). The 3-dimensional maximum intensity projection (MIP) image of the ^18^F-FDG PET/CT showed multiple FDG-avid lesions (Figure 2A). The prostate displayed an unevenly increased FDG uptake, especially on the right side, with an SUV_max_ of 17.6 (Figure 2B). A low-density lesion on the right iliopsoas muscle showed a high FDG uptake with an SUV_max_ of 18.6 (Figure 2C). Multiple osteolytic bone destruction was observed in the spine, bilateral ribs, bilateral iliac bones, and the right acetabulum, with an elevated FDG uptake and an SUV_max_ of 10.3 (Figure 2D). In the abdomen, chest, left armpit, and bilateral neck, multiple lymph nodes showed an increased FDG uptake, with an SUV_max_ of 18.7 (Figure 2E). The ^18^F-PSMA PET/CT demonstrated multiple systemic PSMA-avid lesions on the MIP image (Figure 2F). A slightly unevenly increased PSMA uptake was noted in the prostate, with an SUV_max_ of 3.2 (Figure 2G). In the low-density lesion of the right iliopsoas muscle, the PSMA uptake was slightly unevenly increased and SUV_max_ was 3.1 (Figure 2H). There was a slightly increased PSMA uptake in the bones and lymph nodes (Figures 2I,J).

**Figure 3:**
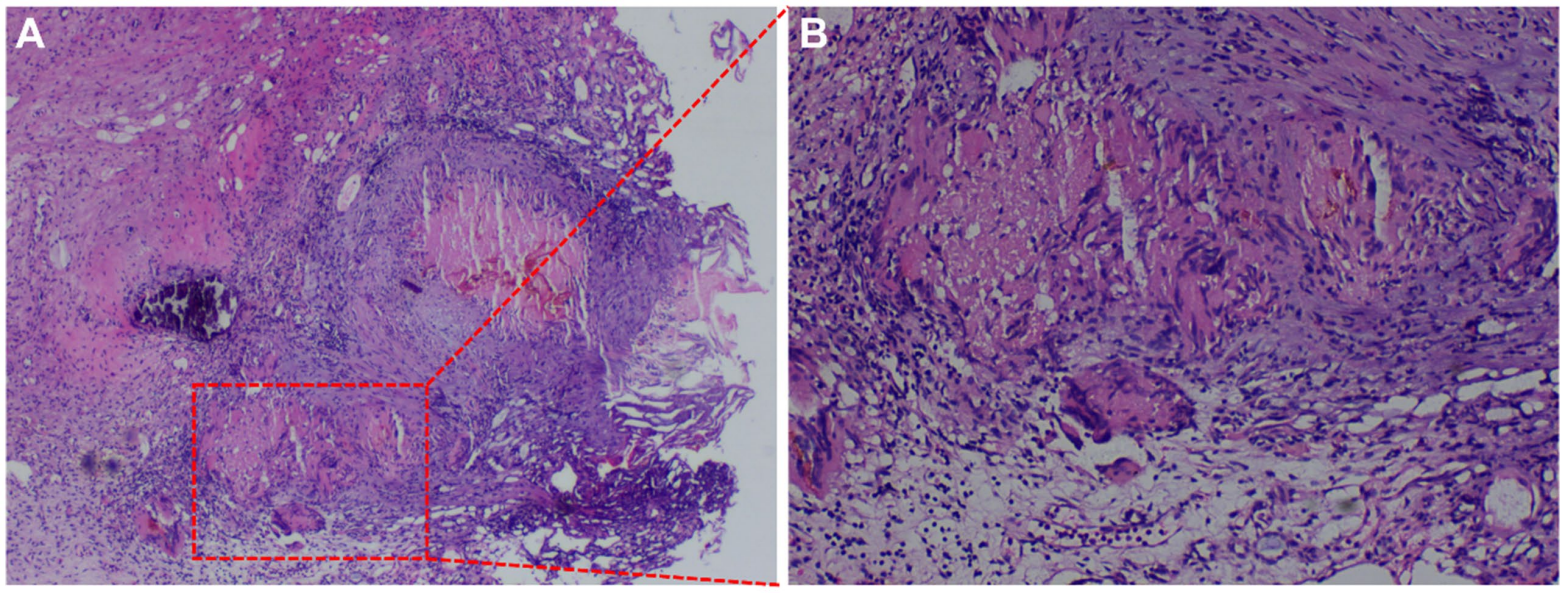
Acid-fast staining of prostate biopsy. **(A)** Multifocal necrosis with granulomatous lesions (×40). **(B)** Multinucleated giant cell reaction (×100).

A transperineal ultrasound-guided (TPUS) 12-core prostate biopsy revealed multifocal necrosis with granulomatous lesions (Figure 3A) and multinucleated giant cell reaction (Figure 3B). Immunohistochemistry of the bilateral internal lower puncture tissues showed CK34βE12 (+), P504S (−), and P63 (+). In addition, based on the positive result of tuberculosis infection of the T cell spot test, systemic tuberculosis was diagnosed. After 6 months of a standard anti-tuberculosis treatment with isoniazid 0.3 g, qd, the patient’s symptoms of dysuria, night sweats, and loss of appetite significantly improved. From the patient’s perspective, he experienced symptom relief, gained a better appetite, and felt less uncomfortable during urination. His overall quality of life had been greatly improved.

## Discussion

Tuberculosis remains a serious public health problem, especially in developing countries. The condition of prostate tuberculosis is an idiopathic infective granulomatous prostatitis, which is often accompanied by both seminal vesicle and epididymal tuberculosis. The prostate is more likely to be affected by hematogenous spread than through the urinary system (4, 5). Prostate tuberculosis is usually asymptomatic, but it can include symptoms such as urinary tract irritation and hematuria. Prostate nodules can be detected during digital rectal examination. At the same time, PSA levels may be normal or elevated, while urine analysis and urine culture are generally negative (6). Cancerous glands lack basal cells, which are found in benign glands. Thus, when confronted with an ambiguous lesion, markers of basal cells (CK34βE12 and P63) are usually used to identify the basal cells (7–9). CK34βE12 and P63 positivity argue against the diagnosis of invasive prostatic carcinoma (9, 10). P504S is a positive marker of prostate carcinoma with high sensitivity (11–13).

Imaging examination plays an important role in prostate tuberculosis. MRI findings of prostate tuberculosis typically show isointensity on T1WI and diffuse or focal hypointensity on T2WI. T2WI of prostate cancer also often shows hypointensity (6). DWI reflects the diffusion ability of water molecules. Due to tight intercellular connections and limited water, prostate cancer typically shows significant limited diffusion or no diffusion and low ADC values. Prostate tuberculosis can also show limited diffusion on DWI or low values on ADC, but the signal is higher than that of prostate cancer, showing significant differences (14). Transrectal ultrasound (TRUS) of prostate tuberculosis has no specific features according to previous literature (6). Hypoechoic lesions, prostate enlargement, and calcification caused by caseous necrosis can be seen. In our study, prostate enlargement with calcification was observed in the patient. Hence, it is often difficult to distinguish prostate tuberculosis from benign prostatic hyperplasia, a prostate malignant tumor, and a urinary tract infection because of their similar clinical and imaging changes. Therefore, we need to enhance the understanding of imaging manifestations of prostate tuberculosis for a better diagnosis in the early stage.

PET is a valuable tool for prognostication and treatment response assessment due to its high sensitivity capabilities for lesion detection at nanomolar concentrations and its quantification capabilities. The most common radiopharmaceutical used in research and clinical practice for PET imaging is FDG, a glucose analogue. However, one major limitation of using FDG is the high false-positive rate due to its poor specificity. Prostatic tissues physiologically express PSMA, a Type II transmembrane protein. However, it is significantly overexpressed by prostate cancer cells (15), and its expression increases with tumor aggressiveness (16). Although PSMA is considered a specific biomarker of prostate cancer, it has been reported that angiogenic factors may stimulate the production of PSMA in tuberculosis tissues. PSMA activity in prostate tuberculosis may partially be attributed to the inflammation caused by infection with *Mycobacterium tuberculosis*, which leads to increased capillary penetration. Physiological reparative reactions within the neovasculature may be another explanation (17). It has been reported that spinal (18), pulmonary (19), and cerebral tuberculosis (17) have an increased uptake of ^68^Ga-PSMA (SUV_max_ 2.3*–*9.6). Compared with latent infection, PSMA avidity may indicate a greater likelihood of active infection (17). In previous studies, PSMA-avid prostate mycobacterial infection has not been reported. In addition, a study by Vorster et al. demonstrated that ^68^Ga-citrate PET/CT is better than FDG PET/CT at imaging pulmonary tuberculosis (PTB) and brain parenchyma tuberculosis (20). Due to the formation of a dense fibrotic cuff around the tuberculosis lesions, radiolabeled inhibitors of fibroblast activation protein (^68^Ga-FAPI) targeting fibrosis have been synthesized for the PET imaging of tuberculosis. Several cases of PTB and extrapulmonary tuberculosis (EPTB), which have an incidental uptake of ^68^Ga-FAPI (21, 22), have been reported.

In this study, the patient had an elevated ESR but a normal PSA value. The prostate lesion manifested a significant increase in the FDG uptake and a slight increase in the PSMA uptake. Multiple osteolytic bone destruction, lymph nodes with an increased FDG uptake, and mildly increased PSMA expression were observed throughout the body. These should be distinguished from the neuroendocrine differentiation in prostate malignant tumors with systemic metastasis, lymphoma, or prostate sarcoma. However, distant lymph nodes are rarely metastatic sites of prostate malignant tumors, and the systemic disease should be considered. A high PSMA uptake in the prostate misled our diagnosis, but the pathological diagnosis was tuberculosis. Indeed, tuberculosis exhibits an increased PSMA uptake under certain circumstances.

## Conclusion

Clinical and imaging manifestations of prostate tuberculosis are atypical. The possibility of tuberculosis should be taken into account in cases where prostate lesions exhibit a high FDG uptake with perilesional abscess, which is rarely observed in metastatic cancer, a mild PSMA uptake elevation, low PSA levels, and pertinent clinical symptoms with infection. Laboratory tests are imperative for further evaluation. However, the ultimate confirmation of the diagnosis still relies on pathological examination.

## Data availability statement

The original contributions presented in the study are included in the article/Supplementary material, further inquiries can be directed to the corresponding author.

## Ethics statement

Written informed consent was obtained from the individual(s) for the publication of any potentially identifiable images or data included in this article.

## Author contributions

XS: Conceptualization, Writing – original draft, Writing – review & editing. YQ: Conceptualization, Data curation, Investigation, Writing – original draft. LS: Formal analysis, Methodology, Software, Writing – review & editing. LK: Funding acquisition, Resources, Supervision, Writing – review & editing.
