# Supplementary figures and images for: Case report: Systemic tuberculosis with prostate involvement mimicking prostate cancer with multiple metastases on 18F-FDG and 18F-PSMA PET/CT
Source: Front Med (Lausanne). 2024 Aug 14;11:1430300. doi: 10.3389/fmed.2024.1430300 (PMC11349513; doi:10.3389/fmed.2024.1430300)

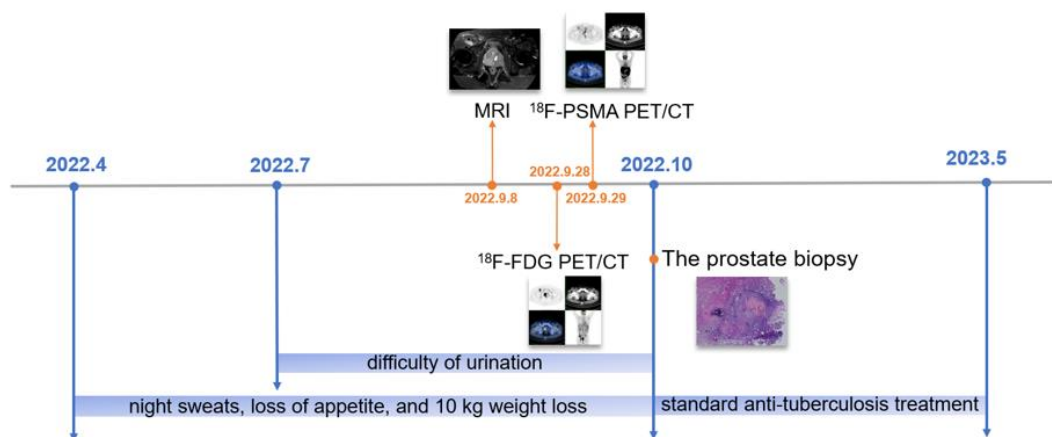

**Figure S1. Timeline.** Important dates and times of the patient in this case.

Supplement: Supplementary file 1 [file Image_1.pdf]
